# Defining Persistent Hotspots: Areas That Fail to Decrease Meaningfully in Prevalence after Multiple Years of Mass Drug Administration with Praziquantel for Control of Schistosomiasis

**DOI:** 10.4269/ajtmh.17-0368

**Published:** 2017-09-25

**Authors:** Nupur Kittur, Sue Binder, Carl H. Campbell, Charles H. King, Safari Kinung’hi, Annette Olsen, Pascal Magnussen, Daniel G. Colley

**Affiliations:** 1 Schistosomiasis Consortium for Operational Research and Evaluation (SCORE), Center for Tropical and Emerging Global Diseases (CTEGD), University of Georgia, Athens, Georgia;; 2 Center for Global Health and Diseases, Case Western Reserve University School of Medicine, Cleveland, Ohio;; 3 National Institute for Medical Research (NIMR), Mwanza Centre, Mwanza, Tanzania;; 4 Parasitology and Aquatic Diseases, Department of Veterinary and Animal Sciences, University of Copenhagen, Copenhagen, Denmark;; 5 Department of Immunology and Microbiology, Centre for Medical Parasitology, University of Copenhagen, Copenhagen, Denmark;; 6 Department of Veterinary and Animal Sciences, Section for Parasitology and Aquatic Diseases, Faculty of Health and Medical Sciences, University of Copenhagen, Copenhagen, Denmark;; 7 Department of Microbiology, University of Georgia, Athens, Georgia

## Abstract

Preventive chemotherapy with praziquantel for schistosomiasis morbidity control is commonly done by mass drug administration (MDA). MDA regimen is usually based on prevalence in a given area, and effectiveness is evaluated by decreases in prevalence and/or intensity of infection after several years of implementation. Multiple studies and programs now find that even within well-implemented, multiyear, annual MDA programs there often remain locations that do not decline in prevalence and/or intensity to expected levels. We term such locations “persistent hotspots.” To study and address persistent hotspots, investigators and neglected tropical disease (NTD) program managers need to define them based on changes in prevalence and/or intensity. But how should the data be analyzed to define a persistent hotspot? We have analyzed a dataset from an operational research study in western Tanzania after three annual MDAs using four different approaches to define persistent hotspots. The four approaches are 1) absolute percent change in prevalence; 2) percent change in prevalence; 3) change in World Health Organization guideline categories; 4) change (absolute or percent) in both prevalence and intensity. We compare and contrast the outcomes of these analyses. Our intent is to show how the same dataset yields different numbers of persistent hotspots depending on the approach used to define them. We suggest that investigators and NTD program managers use the approach most suited for their study or program, but whichever approach is used, it should be clearly stated so that comparisons can be made within and between studies and programs.

## INTRODUCTION

Global efforts to control morbidity due to schistosomiasis were codified by the World Health Assembly (WHA) Resolution 54.19^1^ in 2001. Since then, these efforts have largely rested on preventive chemotherapy (PC) with praziquantel (PZQ), often delivered through mass drug administration (MDA) either in school-based or community-wide programs.^2–4^ These programs have often been implemented by national neglected tropical disease (NTD) control programs partnered with different enabling organizations through bilateral arrangements. As increasing numbers of national NTD control programs have scaled-up MDA, and major PZQ supplies (both donated and purchased) have been announced and provided, the WHA has approved two more resolutions (WHA 65.21 and 66.12) in 2012 and 2014, respectively,^5,6^ that urge programs, where appropriate, to move from the goal of morbidity control to the extended goal of elimination of schistosomiasis as a public health problem, with the eventual objective of interrupting schistosomiasis transmission.

Over the last 15 years, MDA using PZQ has substantially lowered both prevalence and intensity of infection in many areas.^7,8^ However, some subareas, at times as focal as an individual village, fail to substantially decrease their prevalence and mean intensity of *Schistosoma* infections despite several years of well-implemented MDA coverage.^9^ Frequently, it is only when a program or study “goes to scale,” and only if there is sufficiently thorough monitoring, that such focal, village-to-village differences are observable.

The Schistosomiasis Consortium for Operational Research and Evaluation (SCORE; https://score.uga.edu/) has been conducting large-scale operational research into morbidity control. Its aim is to determine the most effective means of providing PC through MDA when delivered either through community-wide treatment (CWT) or through school-based treatment (SBT).^10^ In the performance of these large (75–150 villages) SCORE multiyear studies, we have identified villages that continue to have high prevalence and intensity despite 3 or 4 years of annual MDA. By contrast, other villages, some quite nearby, show reductions to expected low levels of *Schistosoma* infection prevalence and intensity. To better understand the reasons that some villages fail to decline in prevalence and intensity and to determine whether these “persistent hotspots” might be able to be identified after 1 or 2 years of MDA, precise definitions for persistent hotspots need to be applied across all such studies and programs. Clear definitions will be important in considering whether infection in persistent hotspots can be substantially reduced, either by enhancing MDA efforts alone or with the introduction of additional control measures.

The intention of this article is to present possible approaches to operationally define persistent hotspots, using data from one of the SCORE country studies (Tanzania) to illustrate the suggested options. This dataset is representative of the variability of response to annual MDAs that can be seen in multiple other SCORE studies. Their particular variations in MDA response will be addressed in subsequent articles. We discuss the potential use of different definitions for those investigators and NTD program managers who are now seeking to understand and address persistent hotspots. The term, persistent hotspot, as it is used here, requires that locations do not decline as expected in prevalence and intensity in the face of multiyear MDA. This means that by this definition a persistent hotspot is only defined after an intervention; simply starting at high infection prevalence or intensity before control interventions is not sufficient for a location to be termed a persistent hotspot. The examples used in this analysis are from locations that have started with relatively high prevalence. However, other SCORE studies in areas with lower prevalence also demonstrate variability in response to multiyear MDA, and some of the same approaches described could be applied to those areas as they move beyond morbidity control toward elimination of schistosomiasis.

## METHODS

Data from the multivillage SCORE *Schistosoma mansoni* control study based in Mwanza, Tanzania were used to evaluate different approaches to defining persistent hotspot locations observable after 3 years of annual MDA. A description of the overall study, its methods, and baseline data has been published.^10^ Briefly, the Tanzania “Gaining Control” Sm2 study compared annual MDA by CWT and annual MDA by SBT delivered either every year or every other year and was designed to be done in villages with baseline prevalence ≥ 25%. In a few locations, starting prevalence was < 25%. For the purposes of the calculations presented, prevalence and intensity are based on our data from 9- to 12-year-old children, and MDA coverage is defined in both CWT and SBT locations as the proportion of school-aged children (SAC) who had received treatment with PZQ in that round of treatment.

For the purposes of this study, we included only villages or schools in those arms that received MDA every year, whether CWT or SBT, or a serial combination of these two approaches. These correspond to Year 1 and Year 4 data from the 74 villages in Arms 1, 2, and 4 previously described in the protocol paper.^10^ Prevalence and intensity data were collected in cross-sectional testing of 9- to 12-year-old children each year, just before that year’s scheduled MDA.

Year 4 data (collected after 3 years of MDA) were used to assess four possible approaches to defining persistent hotspots. Villages that did not meet the definition for persistent hotspots were said to have had meaningful, operationally acceptable declines in prevalence and/or intensity. As seen below, for the purposes of this exercise, the thresholds used to evaluate the methods described are set arbitrarily. For purposes of our analysis, infection intensity was explored in two ways—1) when calculated among all children who were tested (village-level intensity) and 2) when calculated only for those with *Schistosoma* egg-positive specimens.

### Approach 1. Absolute change in prevalence from Year 1 to Year 4 (prevalence Year 4 minus prevalence Year 1).

Perhaps the simplest approach was based on calculating the absolute change in prevalence rate from before to after intervention, i.e., the starting prevalence minus the prevalence after a given number of rounds of annual MDA. However, because this definition does not adjust for starting prevalence, it masks important differences in the endpoints considered significant. For example, a 20% decrease in prevalence in a location having a starting prevalence of 40% means a decline to 20% prevalence, whereas in a location with a starting prevalence of 80%, a 20% prevalence reduction would result in the community remaining at very high prevalence of 60%.

### Approach 2. Relative percent change in prevalence from Year 1 to Year 4 [(prevalence Year 4 minus prevalence Year 1/prevalence Year 1) × 100].

A second approach would be to determine the relative percent change in prevalence, regardless of starting prevalence. Using this method, the starting prevalence would influence the level of decline, such that going from 80% to 60% would be a 25% drop in prevalence, whereas going from 40% to 20% would be classified as a 50% decline in prevalence.

### Approach 3. Change in World Health Organization (WHO) risk categories (≥ 50%, 10–49%, and < 10% prevalence, as used in their MDA guidelines.

A third approach is to incorporate the current WHO guideline thresholds,^11^ which define prevalence of ≥ 50% as high risk, 10–49% as moderate risk, and < 10% as low risk, for purposes of selecting MDA treatment frequency and coverage in controlling morbidity due to *Schistosoma* infection. By using this method, a “meaningful decline in prevalence” would require prevalence to change from one category to a lower category. Thus, a change after 3 years of MDA from 55% to 45% would be considered a meaningful decline, whereas a community moving from 45% to 15% prevalence would be categorized as a persistent hotspot.

### Approach 4. Combined change in both prevalence and mean intensity of infection from Year 1 to Year 4.

A fourth approach used both prevalence and intensity measurements to define persistent hotspots. In the examples provided, intensity of *S. mansoni* infection refers to a person’s mean eggs per gram of stool measured in standard Kato-Katz microscopy from 3 day’s specimens, two slides per specimen. In the Results, we provide examples using either the absolute changes in prevalence and intensity or the relative changes in prevalence and intensity. These findings are illustrated using a four-quadrant box. The respective quadrants contain locations where 1) the prevalence and mean intensity both increased; 2) the prevalence increased and intensity decreased; 3) the prevalence decreased and intensity increased; and 4) the prevalence and intensity both decreased.

Coverage is important in determining whether a location is a persistent hotspot or the eligible population was simply not adequately treated. Therefore, we conducted our analyses in two ways—the first including all locations and the second including only those locations where adequate coverage was obtained. For the present analysis, adequate coverage in both SBT and CWT villages was defined as ≥ 50% coverage of SAC during the first year of MDA and ≥ 75% in all subsequent years.

The agreement between categorization derived by the different approaches was assessed using kappa statistic. Kappa values were interpreted as poor (< 0.20), fair (0.21–0.40), moderate (0.41–0.60), substantial (0.61–0.80), and perfect (0.81–1.00).^12^

## RESULTS

### Identification of persistent hotspots in the test dataset from SCORE’s study in Tanzania, using each of the four methods considered.

#### Approach 1. Absolute change in prevalence from Year 1 to Year 4 (prevalence Year 4 minus prevalence Year 1).

**Figure 1.**
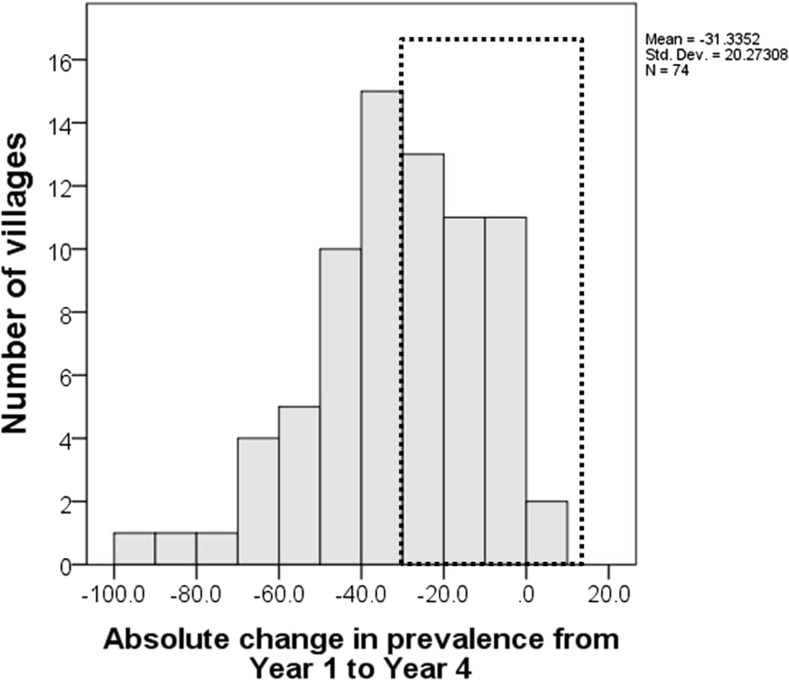
Histogram of the absolute change in *S. mansoni* prevalence after 4 years of mass drug administration in 74 villages in Tanzania. The dotted rectangle indicates persistent hotspots as determined by Approach 1.

When we defined a persistent hotspot as a place with a 30% or less decrease in prevalence, 37 of the 74 schools/villages (50%) met this criterion (Figure 1).

**Figure 2.**
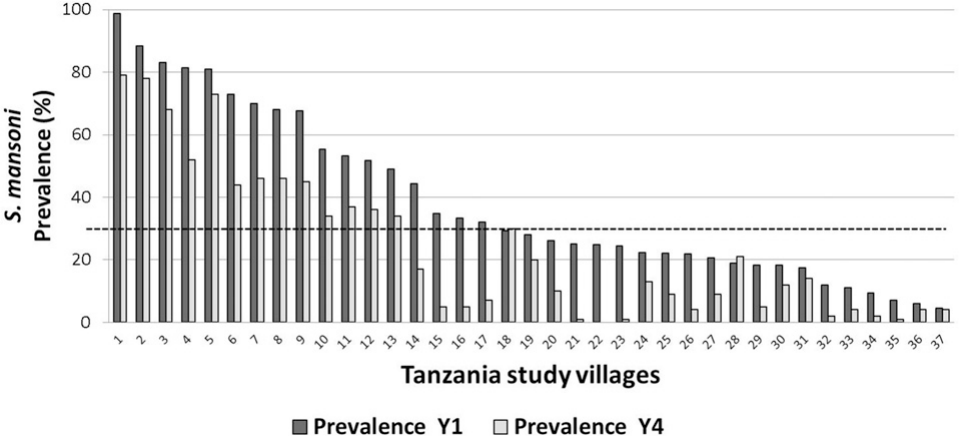
Change in prevalence of *S. mansoni* from Year 1 to Year 4 in the 37 Tanzanian study villages that met the criteria for persistent hotspot using Approach 1. Dashed line indicates 30% prevalence.

Of note, the starting prevalence was > 30% for 17 locations, 10–30% for 16, and < 10% for 4 (Figure 2). It is obvious that a location that started below 30% prevalence could not decrease its prevalence value more than 30% and would be automatically called a persistent hotspot under Approach 1. In Figure 2, the thirty percent prevalence threshold is indicated by the dashed line.

#### Approach 2. Relative percent change in prevalence from Year 1 to Year 4 [(prevalence Year 4 minus prevalence Year 1/prevalence Year 1) × 100].

**Figure 3.**
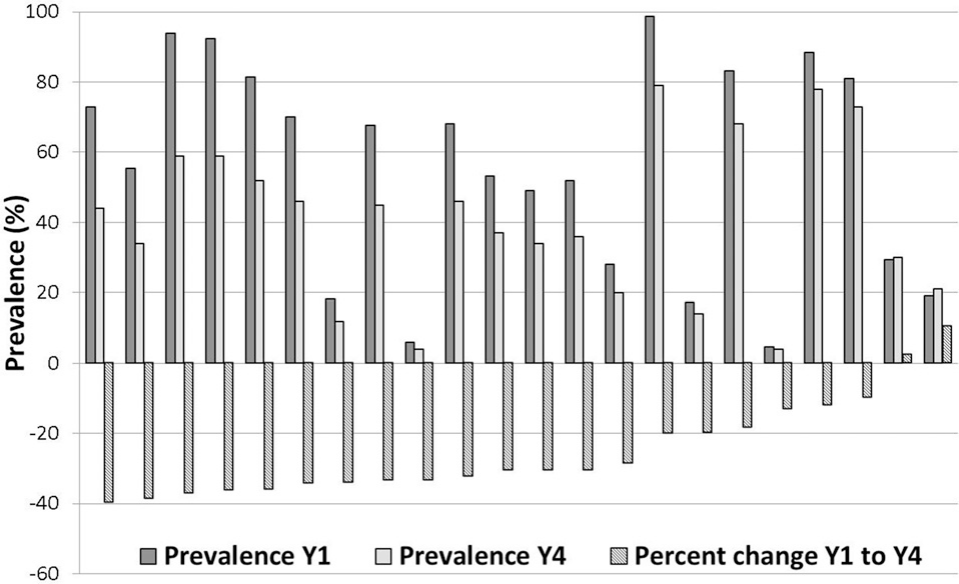
Approach 2 hotspots: Prevalence in Year 4 and Year 1, and percent change in prevalence among Tanzanian villages with < 40% relative decrease in *S. mansoni* prevalence.

For this method, which took starting prevalence into consideration, we chose an arbitrary level of < 40% relative change to define a persistent hotspot. By this approach, 22 locations—30% of the test dataset—were classified as persistent hotspots (Figure 3). Using this method, locations that started out at a low prevalence could either be categorized as persistent hotspots or as adequately responding sites.

Fewer locations were defined as persistent hotspots by Approach 2 as compared with Approach 1 (22 versus 37, respectively). All but two locations that were identified as persistent hotspots by Approach 2 were also identified as persistent hotspots by Approach 1. These two locations both had starting prevalences of more than 90% and decreased to just < 60% by Year 4. So, although the prevalences in these two locations decreased by more than 30% in absolute terms (“adequate” under Approach 1), they remained quite high, even after 3 years of MDA, with less than a 40% relative reduction in prevalence (Approach 2) during intervention.

Seventeen villages that were classified as persistent hotspots by Approach 1 were reclassified as declining by Approach 2. Most of these villages had a starting prevalence of < 30%, which means they had been automatically classified as persistent hotspots under Approach 1.

#### Approach 3. Change in WHO risk categories (≥ 50%, 10–49%, and < 10% prevalence, as used in their MDA guidelines.

The WHO recommended treatment strategy for schistosomiasis for achieving morbidity control classifies communities into high risk (≥ 50%), moderate risk (10–49%), and low risk (< 10%) based on prevalence. For *S. mansoni*-endemic areas, these thresholds are based on infection detection using the Kato-Katz thick smear fecal microscopy assay. In Approach 3, we defined a location that remained within its risk group after several rounds of MDA as a persistent hotspot, whereas movement to a lower risk group classified it as a declining location with adequate response. For example, a location starting at ≥ 50% prevalence that remains at ≥ 50% prevalence would be classified a persistent hotspot, whereas a location that fell to < 50% prevalence would be said to be declining, by virtue of moving to a lower prevalence category. For locations starting at 10–49% prevalence, persistent hotspots would be those that remain at ≥ 10%. Finally, for low-prevalence locations (< 10%), we arbitrarily chose a separate cutoff for response, in which a declining village would be one that fell to < 3% prevalence.

Using Approach 3 to define persistent hotspots for the 74 locations in the dataset, 19 (about a fourth) were classified as persistent hotspots. Of the 43 communities starting in the high-risk category (≥ 50% starting prevalence), 30 (representing 70% of the dataset) changed to a lower-prevalence category and were classified as declining locations. Seven locations (17%) did not change to a lower-risk category (Table 1) and were therefore categorized as persistent hotspots. Of note, a hypothetical location that decreased in prevalence from 50% to just 49% after several years of annual MDA would not be classified as a persistent hotspot using this approach. Of the 27 locations that started as moderate-risk communities, 10 (about a third) were classified by Approach 3 as persistent hotspots, whereas the rest were classified as declining locations. Despite prevalence eligibility surveys having been done before categorization of the communities, four of 74 locations were subsequently found to be low-risk communities at the start of the study and two of those declined to < 3%.

**Table 1:** Classification of 74 Tanzanian villages using the criterion of failure to move to a lower WHO risk category as the definition of a persistent hotspot

		Prevalence in Year 4				Total
		≥ 50%	10–49%	3–< 10%	< 3%	
Prevalence in Year 1	≥ 50%	**7**	30	6	–	43
	10–49%	0	**10**	17	–	27
	< 10%	0	0	**2**	2	4
Total		7	40	25	2	74

Persistent hotspots are indicated in boldface. WHO = World Health Organization.

#### Approach 4. Combined change in both prevalence and village-level mean intensity of infection from Year 1 to Year 4: both as absolute change and percent change.

**Figure 4.**
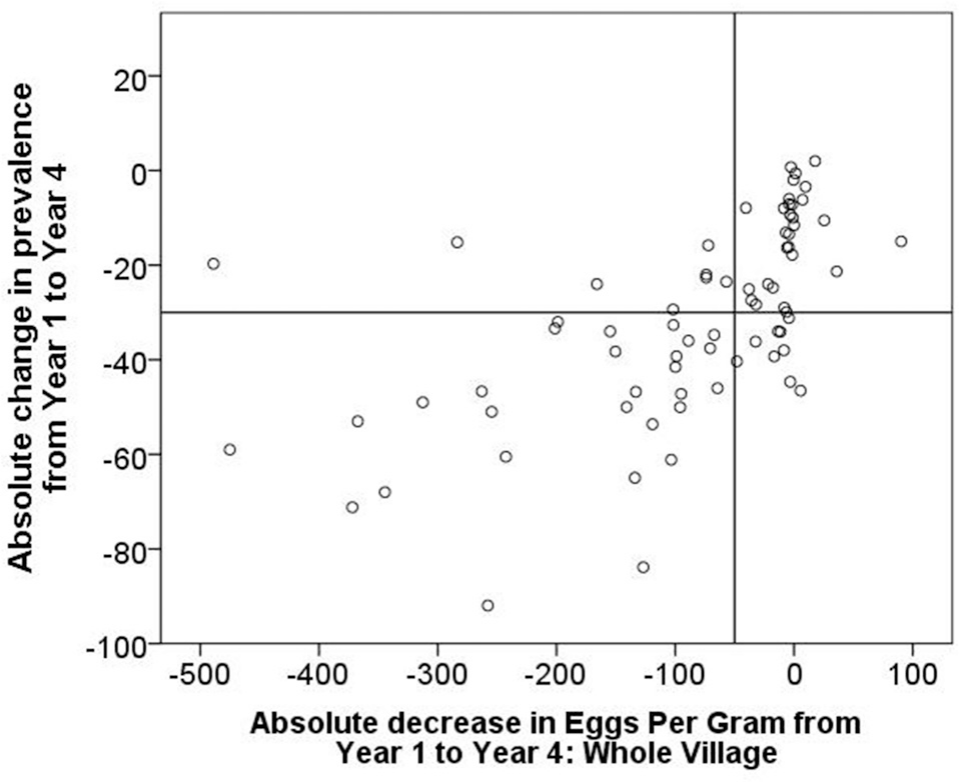
Absolute change in prevalence of *S. mansoni* from Year 1 to Year 4 in 74 Tanzanian study villages, plotted against absolute change in village-level mean intensity (eggs per gram).

**Figure 5.**
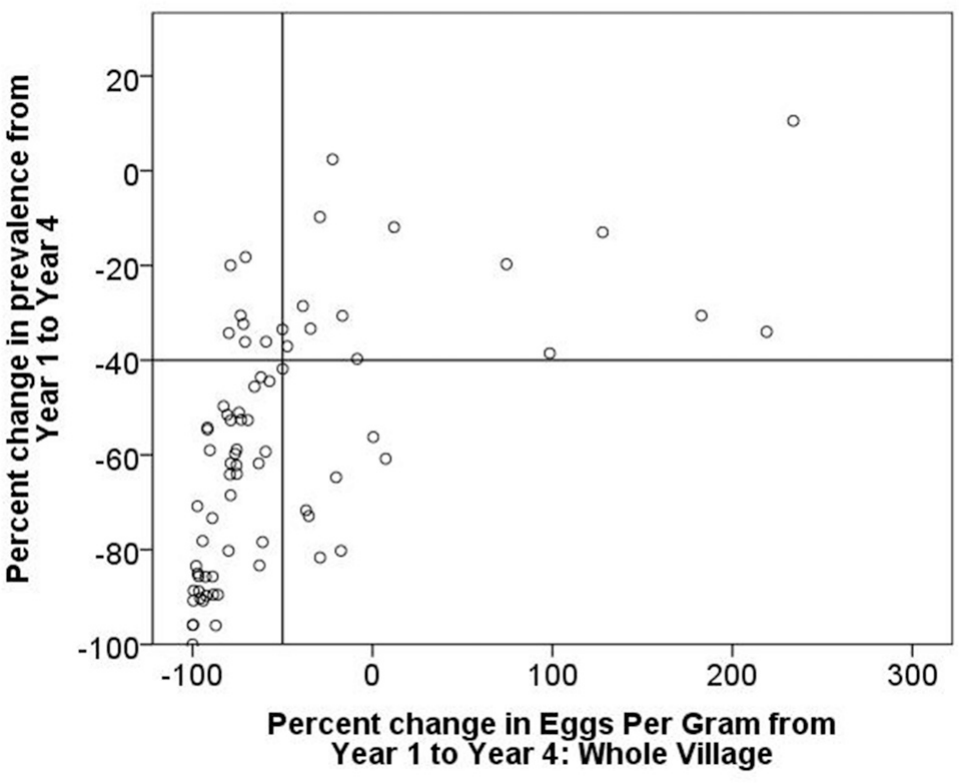
Relative change in prevalence of *S. mansoni* from Year 1 to Year 4 in 74 Tanzanian study villages, plotted against relative change in village-level mean intensity (eggs per gram).

This approach of categorizing persistent hotspots takes into account both the change in prevalence and the change in intensity as determined by the Kato-Katz stool microscopy assay. In this demonstration, we have analyzed both the absolute and the relative (percent) change in prevalence and intensity in Figure 4 and in Figure 5, respectively. By graphing the change in intensity on the *x* axis and the change in prevalence on the *y* axis and then constructing quadrant lines to define arbitrary cutoff points, it is possible to assign treated communities to categorical quadrants to classify how they responded to the intervention by adequately decreasing (or not) in both prevalence and intensity.

We arbitrarily set the quadrant lines in Figure 4 so that a 30% decrease in absolute prevalence (as in Approach 1) and an absolute decrease in village-level mean intensity of 50 eggs per gram would qualify a location as declining (lower left quadrant). Using these cutoffs, 29 locations (39%) fall in the upper-right quadrant and are defined as persistent hotspots. Nine locations (12%) declined more than 30% in prevalence but did not decrease in intensity by 50 eggs per gram of stool (lower right quadrant) and could also be considered as persistent hotspots by this approach although they would be considered declining locations by Approach 1.

When only *S. mansoni* egg-positive study participants were used to calculate mean intensity and the observed changes in intensity, 26 locations (35%) were defined as persistent hotspots compared with the 29 locations using the village-level mean intensity. The latter metric is considered to relate to changes in overall contamination potential within a given village, whereas the former relates to average individual infection levels and may be a better indicator of personal risk for morbidity.

For the figure depicting relative changes in prevalence and intensity (Figure 5), we arbitrarily set the quadrant lines so that a 40% decrease in relative prevalence (as in Approach 2) and a 50% percent decrease in village-level intensity would qualify a location as declining (lower-left quadrant). Using these cutoffs, 14 locations (19%) fall in the upper-right quadrant and are defined as persistent hotspots, as opposed to Figure 4, where using absolute changes classified 29 locations (39%) as persistent hotspots.

Using either approach to assess changes in intensity (Figure 4 or Figure 5), it is clear that where one sets the arbitrary cutoffs for both prevalence and intensity can make a major difference in how locations are categorized, whether as meaningfully declining or as persistent hotspots. A shift in either parameter changes the quadrant-based-assigned categories considerably.

### Comparison of findings using different approaches.

We used kappa statistics to evaluate the agreement among the various response classification approaches that we had developed. Table 2 illustrates concordance between Approaches 1 and 3, and Table 3 illustrates concordance between Approaches 2 and 3.

**Table 2:** Concordance between approach 1 (the absolute change method) and approach 3 (the change in WHO risk category method) in classifying persistent hotspots in 74 villages in Tanzania; Kappa = 0.35

		Absolute change method (approach 1)		Total
		Declining	Persistent hotspot	
WHO risk categories method (approach 3)	Declining	34	**21**	55
	Persistent hotspot	**3**	16	19
Total		37	37	74

WHO = World Health Organization. Villages that were classified as persistent hotspots by one approach and declining by the other approach are indicated in boldface.

**Table 3:** Concordance between approach 2 (the percent change method) and approach 3 (the change in WHO risk category method) in classifying persistent hotspots in 74 villages in Tanzania; Kappa = 0.63

		Percent change method (approach 2)		Total
		Declining	Persistent hotspot	
WHO risk categories method (approach 3)	Declining	48	**7**	55
	Persistent hotspot	**4**	15	19
Total		52	22	74

WHO = World Health Organization. Villages that were classified as persistent hotspots by one approach and declining by the other approach are indicated in boldface.

The kappa statistic measuring the enter-rater agreement between Approach 1 and Approach 3 is 0.35. Thus, there is only fair agreement between these two classification systems. This discordance between Approach 1 and Approach 3 comes about because some locations decreased in prevalence by > 30% but stayed in the same risk category, and conversely, some villages decreased in prevalence by < 30% but changed treatment categories. Some discordance also comes about because villages with a starting prevalence of < 30% are automatically classified as persistent hotspots under Approach 1 regardless of how much their prevalence changed with multiple MDAs.

The comparison of classifications by Approaches 2 and 3 is seen in Table 3, which has a kappa statistic of 0.63, indicating substantial agreement between these two approaches.

The discordance between Approach 2 and Approach 3, although less than between Approach 1 and Approach 3, occurred because some locations showed percent decreases in prevalence of > 40% but remained in the same risk category, and conversely, some locations decreased in prevalence by < 40% but changed risk categories.

### Impact of coverage on designation as persistent hotspot locations.

Poor coverage is likely to be among the first factors to evaluate when some villages fail to decline meaningfully in prevalence. In SCORE studies, adequate coverage was defined as 50% of SAC treated in Y1 and 75% SAC treated in subsequent years, and this is the working definition used in the coverage impact analyses presented below. Table 4 shows that only 29 of the 74 locations in the current dataset achieved adequate coverage by this definition. For Arm 1 (CWT all 3 years), only 4 of the 24 villages were classified as having received adequate coverage.

**Table 4:** Proportion of Tanzanian study villages in study arms 1 (community-wide treatment for 3 years), 2 (community-wide treatment for 2 years followed by school-based treatment for 1 year), and 4 (school-based treatment for 3 years) with adequate and inadequate treatment coverage

		Not adequate (%)	Adequate coverage (%)	Total (%)
Study arm	1	20 (83)	4 (17)	24 (100)
	2	14 (56)	11 (44)	25 (100)
	4	11 (44)	14 (56)	25 (100)
Total		45 (61)	29 (39)	74 (100)

However, there was no correlation in our Tanzania village dataset between having inadequate coverage (by the definition used in these studies: > 50% Year 1 and > 75% subsequent years) and being categorized as a persistent hotspot by Approaches 1, 2, 3, or 4 (Supplemental Tables 1 –5). In fact, many persistent hotspots had adequate coverage, and conversely, many locations that declined in prevalence had “inadequate” coverage, as previously defined. This then points to the local influence of other possible factors, such as those involved in defining the area’s force of transmission, as more likely to be responsible for the variation seen in response to multiple rounds of MDA.

## DISCUSSION

*Schistosoma* transmission and the associated prevalence of schistosomiasis can be highly focal, even in areas considered to be broadly endemic. In many areas at risk, prevalence and intensity levels vary substantially between relatively close village locations.^13^ Such focal distribution is attributed to the nature of the parasite’s life cycle, which requires a concentration of specific vector snails in combination with poor sanitation to maintain continued high levels of transmission.^14^ Despite this well-known problem, many national NTD control programs rely on broad-based implementation of MDA programs with PZQ to control infection. However, as our experience has shown, implementation of a “standard” MDA program can have very mixed results from location to location.

Reliance on MDA for control of morbidity due to schistosomiasis is in large part based on long-standing experience with programs such as that for lymphatic filariasis (LF). In that program, treatment of infection is highly effective in interrupting transmission by the killing of microfilariae. In such cases, focal hotspots are less likely to develop than is the case for schistosomiasis because MDA for LF clearly decreases local LF transmission.^15^ For *Schistosoma* spp*.* infections, human reinfection can occur almost immediately after treatment if a person enters water containing infected snails. This means that although antischistosomal MDA can serve as a form of schistosomiasis morbidity control, its ability to markedly reduce prevalence (when used as a sole intervention) remains dependent on the environmental force of transmission in any given location.

SCORE’s randomized operational research studies on MDA strategies are sufficiently large (25 villages per arm), such that we can compare not only the treatment arms but also examine individual village outcomes within arms. After three or four annual MDAs, our data show that not all locations within an arm demonstrate the expected decreases in prevalence and intensity. We have termed these locations persistent hotspots. However, there are multiple potential ways that might be used to define what constitutes a persistent hotspot. For our purposes, we require a persistent hotspot to be a location that has had multiple MDAs, as distinct from a location that simply had high prevalence before intervention.

Clear definitions will be essential for operational research on persistent hotspots, both to try to determine why they exist and to design and evaluate strategies to “break” their apparent resistance to control. Likewise, such definitions will be critical for NTD program managers to help them achieve the goals of their programs, and to help them reallocate their resources to address persistent hotspots while reducing inputs in locations that have been more responsive.

The analyses presented here show that the approach chosen to define persistent hotspots and the thresholds used in the context of these approaches can greatly influence the number of persistent hotspots identified. For example, in Approach 3, rather than using the current WHO risk categories of ≥ 50–10% one might consider ≥ 50–25% and ≥ 25–10% as break points to delineate persistent hotspots of acceptable declines due to 3 years of MDA, thereby changing the designation of persistent hotspots considerably in some settings. Likewise, the current presentations are based on results after 3 years of annual MDA. Different results might be likely if calculations were based on monitoring after only one or two MDAs. Besides the number of years of treatment, year-to-year variations in transmission patterns, for example due to flooding or drought, can also have an impact. The year-to-year variation is likely to be greater on prevalence than intensity because infection with the few worm pairs needed to result in a prevalent case would likely occur sooner than the time it generally takes to accumulate enough worms to result in a heavy infection.

In general, our preferences are for the use of Approaches 2 and 4, and this is based on Approach 2 taking starting prevalence into account, whereas Approach 4 allows parallel consideration of intensity data when this parameter is available. However, if the starting prevalence is very similar among all targeted locations, Approach 1 may be adequate in defining the persistent hotspots. Approach 3, which is based on current WHO guidelines, is problematic. Perhaps on their revision, Approach 3 can be modified accordingly.

The goal of the current exercise is not to advocate for a specific definition of a persistent hotspot. It is possible that different definitions will be developed in different contexts. We have presented several possible ways to define these, including an evaluation of how applying different definitions might influence the evaluation of program impact. We did not extensively explore the effects of varying prevalence and intensity cutoffs, but it would be easy to use our data to evaluate the impact of choosing different cutoffs from the ones we have selected. Whatever approach is used, it is critical that the user clearly define the approach and cutoffs being used for analysis. Only in that way can results of studies and program performance be compared.

Another consideration when dealing with locations with differences in prevalence and intensity is the sensitivity of the mapping assays used to determine these parameters. For example, the studies in Tanzania used Kato-Katz results to assess prevalence. This test has low sensitivity in areas with low prevalence. Were infection status measured with a more sensitive assay, cutoffs used to define persistent hotspots would need to be changed. For example, an assay with low sensitivity will underestimate the true prevalence achieved when moving from high prevalence to low prevalence, resulting in categorizations as persistent hotspots. Thus, as the sensitivities of assays improve new thresholds will need to be defined.

The results from our assessment of using coverage in defining persistent hotspots are counter-intuitive. One would expect that if a village does not have adequate coverage, it would likely be a persistent hotspot. However, in the dataset used in this study, low coverage by the study definition was substantial (most such villages hovering around 50% the first year and just below 75% in subsequent years). The analyses indicate that at this level, coverage was not the sole or primary defining predictor of being a persistent hotspot. This, of course, does not mean that programs should not strive for the best coverage they can achieve. Certainly, if programs have universally poor coverage, one would expect there to be many more and perhaps universal persistent hotspots. Our analyses, however, focus attention on the importance of very local factors in the transmission of schistosomiasis. Our working hypothesis is that a persistent hotspot is a location that has a high force of transmission, and thus, reinfection reliably occurs after MDA. By contrast, we hypothesize that a location that responds well by steadily declining in prevalence would be where MDAs appreciably lower the force of transmission, slowing the rate of reinfection.

Identification of the factors responsible for the genesis of persistent hotspots is the topic of two new SCORE studies. Potential factors under consideration are general terrain; low level of sanitation; limited use of sanitation; limited availability of clean water; amount or seasonality of surface water; water usage (agriculture/household); number, type, and location of water contact sites; local abundance of snail habitat; snail strains and their relative susceptibilities; genetic variation among schistosomes; the presence of competitor or hybrid schistosomes; genetic variation among exposed people; variable drug effectiveness and variable levels of compliance in taking the drug; and persistence of transmission because of systematic noncompliance. The force of transmission is clearly determined by multiple factors that in concert do or do not facilitate reinfection after treatment.

In addition to accurately defining persistent hotspots and attempting to identify the factors that contribute to their existence, there is an urgent need to determine how an NTD program manager can successfully deal with them when they occur. Defining what interventions can change a persistent hotspot into one that is meaningfully declining will next require additional operational research. Possible approaches include increases in PC, use of better drugs or drug combinations, inclusion of snail control measures, and the application of sanitation and the provision of clean water. However, in the short term, many of these are not feasible or are not supported politically.

Currently, it is not standard practice to monitor the impact of schistosomiasis MDA programs on a frequent basis. However, data from large studies such as ours clearly indicate that response to MDA can vary considerably among nearby villages. It would be beneficial to identify those locations that are persistent hotspots as early as possible, especially if it would allow more intensive, targeted efforts in those locations. Such evaluations would, in parallel, identify places that have responded sufficiently to MDA, and might therefore require less intensive efforts. However, at this time it seems such characterizations can only be made after at least some MDA. With the use of new point-of-care mapping tools, it may be possible to move to more frequent monitoring and thus detect persistent hotspots sooner. Being able to empirically define persistent hotspots would hopefully ensure that resources for more intensive intervention are distributed in locations that need them the most.

The current study was based on data from the SCORE study in Mwanza, Tanzania. This is a generally high-prevalence setting. The same pattern of variable response to MDA is apparent in all the multiyear randomized SCORE trials, including in those starting at low prevalence levels.^9^ Developing strategies for defining, identifying, and addressing persistent hotspots for places with a broad range of prevalence rates will be critical if morbidity control and eventually elimination is to be achieved.

## Supplementary Material

Supplemental Table.
