# Supplementary material for: Defining Persistent Hotspots: Areas That Fail to Decrease Meaningfully in Prevalence after Multiple Years of Mass Drug Administration with Praziquantel for Control of Schistosomiasis
Source: Am J Trop Med Hyg. 2017 Sep 25;97(6):1810–7. doi: 10.4269/ajtmh.17-0368 (PMC5805060; doi:10.4269/ajtmh.17-0368)
Supplement: Supplementary file 1 [file tpmd170368.SD1.pdf]

Supplemental Table 1

Correlation between having attained adequate coverage and meeting criteria for adequate response (nonhotspot), as defined by the absolute change in prevalence (approach 1); χ^2 $P = 0.096$

		Not adequate coverage (%)	Adequate coverage (%)	Total (%)
Absolute change approach	Declining	19 (51)	18 (49)	37 (100)
	Persistent hotspot	26 (70)	11 (30)	37 (100)
	Total	45 (61)	29 (39)	74 (100)

Supplemental Table 2

Correlation between having attained adequate coverage and meeting criteria for adequate response (nonhotspot) as defined by the percent change in prevalence (approach 2); χ^2 $P = 0.172$

		Not adequate coverage (%)	Adequate coverage (%)	Total (%)
Percent change approach	Declining	29 (56)	23 (44)	52 (100)
	Persistent hotspot	16 (73)	6 (27)	22 (100)
	Total	45 (61)	29 (39)	74 (100)

Supplemental Table 3

Correlation between having attained adequate coverage and meeting criteria for adequate response (nonhotspot) as defined by the change in WHO risk categories (approach 3); χ^2 $P = 0.431$

		Not adequate (%)	Adequate coverage (%)	Total (%)
WHO risk categories approach	Declining	32 (58)	23 (42)	55 (100)
	Persistent hotspot	13 (68)	6 (32)	19 (100)
	Total	45 (61)	29 (39)	74 (100)

WHO = World Health Organization.

Supplemental Table 4

Correlation between having attained adequate coverage and meeting criteria for adequate response (nonhotspot) as defined by the absolute change in both prevalence and intensity (approach 4); χ^2 $P = 0.257$

		Not adequate (%)	Adequate coverage (%)	Total (%)
Absolute prevalence + absolute village-level intensity approach	Prevalence decreased ≥ 30%, intensity decreased ≥ 50 epg (declining)	13 (46)	15 (54)	28 (100)
	Prevalence decreased ≥ 30%, but intensity decreased < 50 epg	6 (67)	3 (33)	9 (100)
	Prevalence decreased < 30%, intensity decreased ≥ 50 epg	6 (75)	2 (25)	8 (100)
	Prevalence decreased < 30%, intensity decreased < 50 epg (persistent hotspots)	20 (69)	9 (31)	29 (100)
	Total	45 (61)	29 (39)	74 (100)

Supplemental Table 5

Correlation between having attained adequate coverage and meeting criteria for adequate response (nonhotspot) as defined by the relative change in both prevalence and intensity (approach 4); χ^2 $P = 0.274$

		Not adequate (%)	Adequate coverage (%)	Total (%)
Relative changes in prevalence + village-level intensity approach	Prevalence decreased ≥ 30%, intensity decreased ≥ 50 epg (declining)	23 (52)	21 (48)	44 (100)
	Prevalence decreased ≥ 30%, intensity decreased < 50 epg	6 (75)	2 (25)	8 (100)
	Prevalence decreased < 30%, intensity decreased ≥ 50 epg	5 (63)	3 (37)	8 (100)
	Prevalence decreased < 30%, intensity decreased < 50 epg (persistent hotspots)	11 (79)	3 (21)	14 (100)
	Total	45 (61)	29 (39)	74 (100)
